# Medical Monopoly

**Published:** 1892-09

**Authors:** 


### MEDICAL MONOPOLY.

(Editorial in September number of *The Medical Visitor.*)

Monopoly, in whatever shape presented, is abhorrent to the American people, and especially to those residing in the generous, open-hearted West, where fair play is ever the motto. What, then, shall be said of the recent attempt of the Illinois State Board of Health to establish a medical monopoly?

*The Medical Visitor*, which is interested in all worthy colleges and connected with none, has had occasion to criticise the Board in the past, and is forced to do so again after receiving the minutes of the meeting of July 27th. It seems, although the Board does not say so, that the intention is to cripple the University of Chicago, that splendid institution which has already spent millions, and which was about to establish a medical department. As this would injure some or all of the colleges with which the members of the Board are connected, especially as the University, it is said, had declined to take any of them under its wings, the institution must be throttled in some way, and what better way than to require that all of its students, or in fact, the students of any new institution in the United States, must be examined by this Board, composed of members with the souls of pigmies. No college, says the Board, of less than five years' existence will be recognized. However, the poor graduate can come before the Board, and if the feeling of hatred is not too intense he will graciously be allowed to slip through.

With such a Board in existence all medical education and progress not only in this State, but in the United States, must come to a standstill until the courts again clip the wings of this useless and obnoxious Board. In every instance in which this body has resorted to the courts it has been beaten, and certainly will be again when the time comes for this question to be decided. It is only necessary to refer to Judge McAlister's decision, well known to the physicians of this State. Certain police powers were granted to this Board, and now these policemen, "puffed up with a little brief authority," would try to regulate Mars, if they could only reach it.

Class legislation is no more popular than monopoly, and if the Board desires to examine only the graduates of new institutions, like the University of Chicago and the National Homœopathic Medical College, because they have not been in existence five years, it is class legislation of the rankest kind. Let the Board examine all graduates or none. The Lord only knows how many men from the colleges these professors are connected with could pass muster. It would be a wise act on the part of the Governor to remove these small-souled, narrow-minded bigots from the Board, for any man who will resort to such a despicable method to build up his own college, and attempt to kill general medical education and progress is unfit and unworthy the position.

